# Formation of global self-beliefs in the human brain

**DOI:** 10.1073/pnas.2003094117

**Published:** 2020-10-15

**Authors:** Marion Rouault, Stephen M. Fleming

**Affiliations:** ^a^Wellcome Centre for Human Neuroimaging, University College London, London WC1N 3AR, United Kingdom;; ^b^Department of Cognitive Studies, Ecole Normale Supérieure, Paris Sciences et Lettres University, 75005 Paris, France;; ^c^Max Planck University College London Centre for Computational Psychiatry and Ageing Research, University College London, London WC1B 5EH, United Kingdom;; ^d^Department of Experimental Psychology, University College London, London WC1H 0AP, United Kingdom

**Keywords:** confidence, metacognition, fMRI, self-efficacy

## Abstract

Humans create metacognitive beliefs about their performance across many levels of abstraction—from local confidence in individual decisions to global estimates of our skills and abilities. Despite a rich literature on the neural basis of local confidence judgements, how global self-performance estimates (SPEs) are constructed remains unknown. Using functional magnetic resonance imaging, we scanned human subjects while they performed several short blocks of tasks and reported on which task they think they performed best, providing a behavioral proxy for global SPEs. In a frontoparietal network sensitive to fluctuations in local confidence, we found that activity within ventromedial prefrontal cortex and precuneus was additionally modulated by global SPEs. In contrast, activity in ventral striatum was associated with subjects’ global SPEs irrespective of fluctuations in local confidence, and predicted the extent to which global SPEs tracked objective task difficulty across individuals. Our findings reveal neural representations of global SPEs that go beyond the tracking of local confidence, and lay the groundwork for understanding how a formation of global self-beliefs may go awry in conditions characterized by distorted self-evaluation.

## Significance

Momentary feelings of confidence accompany many of our actions and decisions. In addition to such “local” feelings of confidence, we also construct “global” confidence estimates about our skills and abilities (global self-performance estimates or SPEs). Distorted SPEs may have a pervasive impact on motivation and self-evaluation, for instance affecting estimates of our competitiveness at work or in a sports team. Here, we found that components of a brain network previously implicated in the tracking of local confidence was additionally modulated by SPE level, whereas ventral striatum tracked SPEs irrespective of confidence. Our findings of a neurocognitive basis for global SPEs lay the groundwork for understanding how distorted SPEs arise in educational and clinical settings.

Humans have an ability to internally evaluate the success or failure of other cognitive processes, a capacity known as metacognition (1). In the context of decision-making, metacognition is especially important for behavioral adaptation when external feedback is absent—a situation often encountered in daily life (2, 3). However, a major determinant of human behavior is not only “local” confidence estimates in isolated decisions but also an overall sense of confidence about our abilities, which we refer to as global self-performance estimates (SPEs) (3–5). For instance, in forming a global SPE about our job performance, we might integrate multiple local confidence estimates about various independent decisions and tasks that we have carried out. These global SPEs (which may be related self-efficacy estimates) are thought to determine the goals we choose to pursue and regulate the motivation and effort put into pursuing them (6, 7). Conversely, overinflated SPEs may lead to overconfidence and risky decision-making (8). A key insight of previous work on self-efficacy is that global SPEs can dissociate from objective abilities, and distorted SPEs are linked to a number of mental health symptoms (9–11). However, in contrast to a substantial literature on the neural substrates of local confidence, the computational and neural bases of global SPEs remain poorly understood.

Initial work on the neural underpinnings of metacognition has focused on frontoparietal brain networks underlying the formation of local confidence, i.e., confidence in individual decisions (12, 13). For instance, the dorsal anterior cingulate cortex (dACC) has been identified as a key center for performance monitoring and error detection (14–18), whereas ventromedial prefrontal cortex (vmPFC) and adjacent perigenual anterior cingulate cortex (pgACC) positively track local confidence across multiple cognitive domains (19), including perception (20, 21) and value-based choices (22, 23). In addition, local confidence in mnemonic (24) and perceptual (25) judgments has been associated with activity profiles of the precuneus (PRECU) situated in medial parietal cortex. Taken together, these findings provide converging evidence that midline frontoparietal regions vmPFC, PRECU, and dACC serve as key hubs for confidence formation in the human brain, which, together with anterior prefrontal regions implicated in self-reflection (26, 27), may support a large range of metacognitive abilities.

We have recently obtained behavioral evidence that human subjects build global SPEs by learning from local confidence over multiple instances when external feedback is withheld (3). Previous studies have found that local confidence fluctuations play a role in learning (2, 28), but it remains unknown how these signals interface with global representations of self-performance. Here, human subjects underwent functional magnetic resonance imaging (fMRI) while performing miniblocks of two perceptual tasks. At the end of each block, they were asked to choose the task on which they thought they performed best. These task choices constituted our proxy for assaying global SPEs. By sorting trials according to whether they correspond to higher or lower global SPEs (tasks that were chosen or not at the end of the block), we identified activity profiles in vmPFC and PRECU in which local confidence encoding was modulated by global SPE. Conversely, activity in ventral striatum was related to shifts in overall global SPEs, irrespective of local confidence. Our findings provide initial evidence for the coexistence of local and global metacognitive representations in the human brain.

## Results

### Experimental Paradigm Assessing the Formation of Global SPEs.

**Fig. 1.**
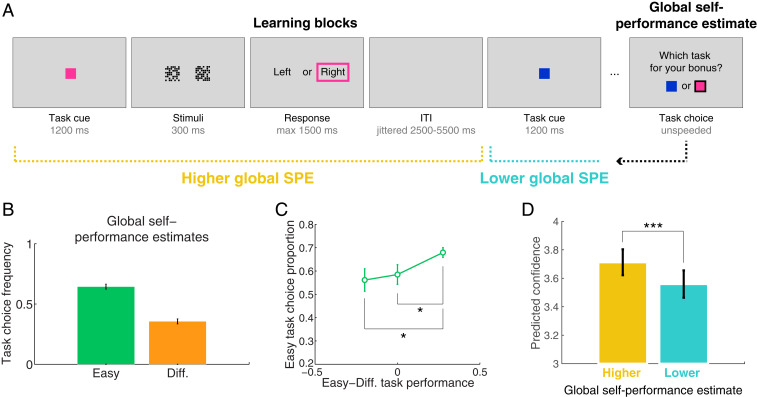
Experimental design and subjects’ behavior (*n* = 41). (*A*) Participants performed short blocks of two distinct perceptual tasks, signaled by a color cue at the beginning of the trial. Both tasks involved decisions about which of two boxes contained more small squares. Participants received no feedback about their judgments. At the end of blocks, subjects were asked to choose between the two tasks for a monetary bonus that was maximized when they chose the task on which they performed best (*Materials and Methods*). These task choices constituted our proxy for global self-performance estimates (SPEs). A posteriori, we labeled each trial as corresponding to a higher or a lower global SPE according to end-of-block task choices (*Results*). Color cues were reset on each new block. (*B*) Easy tasks were selected more often than difficult (Diff.) tasks at the end of blocks, consistent with subjects forming higher global SPEs for easier tasks. (*C*) Global SPEs were also sensitive to both objective difficulty level and fluctuations in performance. *P < 0.034, paired *t* tests. (*D*) Model-derived confidence displayed on a 1 (low) to 6 (high) scale was more elevated on tasks with higher (yellow) than lower (blue) global SPEs in the fMRI experiment. ***P < 0.000001, paired *t* test. Error bars are SEM across subjects.

To probe the construction of global SPEs, our experimental design featured short miniblocks in which subjects played trials of two tasks in random alternation. Every task required a series of perceptual decisions about which of two images contained more small squares (Fig. 1*A*). No feedback was provided, such that subjects could only rely on internal evaluations when forming global SPEs. In any given block, one task was easy and the other one was difficult, as manipulated by the difference in number of small squares between images based on a calibration procedure performed beforehand (*Materials and Methods*). At the end of each block, subjects were incentivized to choose the task on which they thought they performed best, providing a behavioral proxy for global SPEs (3). A new block then ensued with two new color cues indicating two new tasks. To examine how fluctuations in local confidence relate to global SPEs, we labeled each trial a posteriori as belonging to the task that correspond to a higher (chosen) or a lower (unchosen) global SPE, as revealed by end-of-block task choices (Fig. 1*A*).

### Global SPEs Are Sensitive to Local Task Difficulty and Performance.

Subjects’ performance was significantly higher on easy (88% correct) than difficult (72% correct) tasks (*t*_40_ = 23.6, *P* < 1e-20) (*SI Appendix*, Fig. S1*A*). This difference was mirrored in response times (RTs) with subjects responding faster on easy (mean, 799 ms) than difficult (mean, 852 ms) tasks (*t*_40_ = 11.4, *P* < 1e-13), confirming that our difficulty manipulation based on an individual calibration procedure was effective (*SI Appendix*, Fig. S1*B*). We next turned to our main measure of interest—global SPEs—as assayed by end-of-block task choices. As expected, subjects chose easy tasks more often than difficult tasks (on 64% of blocks on average; Fig. 1*B*). Fluctuations in performance within a given difficulty level also modulated global SPEs, indicating that subjects were sensitive to variations in their own accuracy from block to block (Fig. 1*C*). Specifically, in blocks where the objective performance in both tasks was equal, easy and difficult tasks were chosen almost equally often (one-sample *t* test against equal frequency of 0.5: *t*_38_ = 2.003, *P* = 0.052; Bayesian one-sample *t* test Bayes factor [BF] = 1.042, indicating no evidence either for or against a difference from 0.5) (Fig. 1*C*). In contrast, when the difference in performance between tasks was larger, the easy task was chosen more frequently than the difficult one (frequency of choosing the easy task choice when the difference in performance between tasks was positive vs. negative: *t*_38_ = 2.51, *P* = 0.016; was positive vs. null: *t*_38_ = 2.206, *P* = 0.034). A logistic regression predicting task choice confirmed a significant contribution of both difficulty level (*β* = 0.405, *P* = 4.9e-09) and objective accuracy (*β* = 0.35, *P =* 1.7e-06) in the absence of an interaction between these factors (*β* = −0.05, *P* = 0.37).

In summary, both objective difficulty level and local fluctuations in performance informed subjects’ global SPEs, replicating our previous findings (3). Importantly, however, substantial variability in subjects’ task choices remained. Subjects did not always select the easiest of both tasks (range, 38–91% of blocks across subjects), and did not always choose the task on which they performed best (range, 62–96% of blocks across subjects). This partial dissociation between experimental variables and subjective SPEs ensured that our analysis of neural underpinnings of global SPEs was not systematically confounded with difficulty level or objective performance.

### Inferring Local Confidence in the fMRI Experiment.

To investigate activity tracking local confidence in the absence of explicit confidence ratings in the scanner, we next developed a subject-specific model of confidence. Note that we avoided collecting trial-by-trial confidence reports in the fMRI experiment so as not to interrupt the process of learning global SPEs with additional task requirements (*Materials and Methods* and *SI Appendix*). Instead, we leveraged a metacognition task that subjects completed afterward, in which subjects gave explicit local confidence ratings for similar perceptual judgements to those encountered in the fMRI experiment.

A detailed analysis of participants’ behavior in the metacognition task is provided in *SI Appendix*. In brief, we built a subject-specific regression model predicting trial-by-trial confidence reports from other trial-specific behavioral variables (including accuracy, RT, and difficulty) recorded during the metacognition task, and applied these regression coefficients to behavior recorded during the fMRI experiment to infer a proxy for local confidence (*Materials and Methods* and *SI Appendix*, Fig. S2). We established that inferred confidence in the fMRI experiment was higher for easy than difficult trials, and higher for correct than error trials, as expected (*SI Appendix*, Fig. S2*D*). Critically, we also found that the mean predicted local confidence was higher on trials corresponding to higher as compared to lower global SPEs in the fMRI experiment (paired *t* test, *t*_40_ = 6.40, *P* = 1.4e-07) (Fig. 1*D*), in line with our previous analyses of explicit confidence judgments recorded in a similar task (3). Accordingly, a logistic regression explaining task choices revealed a significant contribution of the difference in predicted confidence between tasks (median regression coefficient across subjects, 0.84; one-sample *t* test over regression coefficient, *t*_40_ = 7.3, *P* = 7.1e-09), further supporting the reliability of our approach for inferring local confidence in the fMRI experiment.

We next asked whether our latent confidence model was a better predictor of global SPEs than behavioral variables alone. We found that both the difference in accuracy between tasks (*t*_40_ = 7.5, *P* = 4.2e-09) and the difference in RT between tasks (*t*_40_ = −6.7, *P* = 4.5e-08) separately predicted global SPE choices. To quantify the advantage of the local confidence model over models containing only these components, we computed the sum of deviance (a measure of goodness of fit; *Materials and Methods*) across subjects. This term was 1,538.0 for the predicted confidence model, 1,607.7 for the accuracy model, and 1,585.4 for the RT model, indicating that our confidence estimate was a better predictor of global SPE choices than accuracy or RTs alone. In summary, we replicate previous findings that subjects’ global SPEs are sensitive to local fluctuations in confidence and accuracy (3).

### Analysis of Brain Activity Sensitive to Local Confidence.

**Fig. 2.**
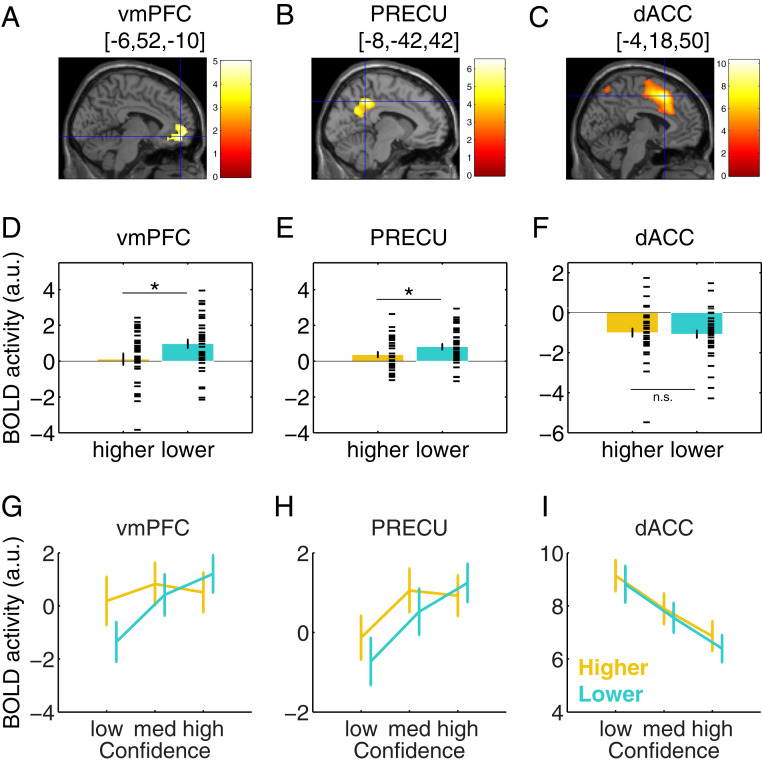
ROI analysis of interactions between local confidence and global SPEs. Local confidence-related activity in ventromedial prefrontal cortex (vmPFC) and a precuneus cluster (PRECU) differed between trials that correspond to higher and lower SPEs as revealed by end-of-block task choices. (*A*–*C*) Clusters in vmPFC (*A*), PRECU (*B*), and dACC (*C*) showing main effects of local confidence (*P* < 0.05, FWE cluster-corrected at a cluster-defining threshold of *P* = 0.001, uncorrected) superimposed on an anatomical template. MNI coordinates of peak activity are indicated in brackets. The color bar indicates the *t* value. (*D*–*F*) Regression coefficients within vmPFC (*D*), PRECU (*E*), and dACC (*F*) clusters (extracted using a leave-one-out procedure from the corresponding statistical maps in *A*–*C*) for the effects of local confidence separately for higher and lower global SPEs. Horizontal ticks indicate individual data points. *P < 0.05, n.s. nonsignificant, paired *t* tests. (*G*–*I*) Visualization of activity profiles corresponding to the effects reported in *D*–*F* (*Materials and Methods*). “Low,” “med,” and “high” correspond to bins of low, medium, and high local confidence. Error bars represent SEM across subjects (*n* = 39). See also *SI Appendix*, Table S1 and Fig. S5.

**Fig. 3.**
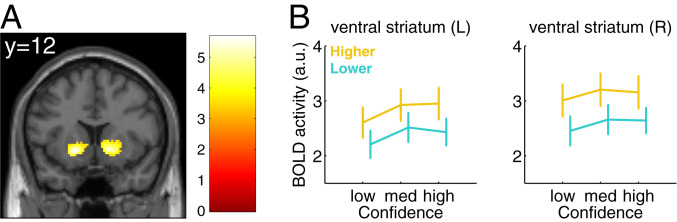
Main effect of global SPEs on brain activity, irrespective of local confidence. (*A*) When contrasting trials that correspond to higher vs. lower global SPEs, we identified increased activity in bilateral ventral striatum and a number of occipital areas (not shown), thresholded at *P* < 0.05 FWE cluster-corrected at a cluster-defining threshold of *P* = 0.001, uncorrected (*Materials and Methods*). *y* indicates the MNI coordinate of the slice. The color bar indicates the *t* value. (*B*) Activity in left (L) and right (R) ventral striatum ROIs for bins of local confidence, with “low,” “med” (medium), and “high” indicating levels of local confidence, for trials corresponding to higher (yellow) and lower (blue) global SPEs. Error bars represent SEM across subjects (*n* = 39).

We next turned to our fMRI data to interrogate the neural underpinnings of the formation of global SPEs. Our initial strategy for analyzing activations focused on whether and how local confidence activity is modulated by the current global SPE. Predicted confidence was entered as a parametric regressor modulating blood oxygen level-dependent (BOLD) signal at response onset, and each trial was labeled as corresponding to a higher or a lower global SPE as revealed by end-of-block task choices (*Materials and Methods*). This analysis approach allowed us to first identify a network of regions tracking local confidence, and within this network, ask whether a subset of these activations were sensitive to information about global SPEs (Fig. 2). Finally, we directly contrasted trials corresponding to higher vs. lower SPEs to reveal brain areas sensitive to global SPEs irrespective of local confidence (Fig. 3).

First, we sought to identify whole-brain activity tracking local confidence. To factor out the influence of global SPEs in this analysis, we collapsed over higher and lower SPE regressors. Consistent with previous studies, we identified a number of areas in which activity was modulated by local confidence (Fig. 2 and *SI Appendix*, Fig. S4 and Table S1), including negative correlations within a network that encompassed dACC, presupplementary motor area, inferior frontal gyrus, and bilateral insula (all *β* less than −0.49, all *P* < 0.0006, familywise error [FWE]-corrected for multiple comparisons at the cluster level), and positive correlations in vmPFC, PRECU, fusiform gyrus, bilateral inferior frontal cortex, angular gyrus, and inferior/midtemporal gyrus (all *β* > 1.08, all *P* < 0.0159, FWE-corrected for multiple comparisons at the cluster level) (*SI Appendix*, Fig. S4 and Table S1).

### Local Confidence Activity Sensitive to Global SPEs.

We next turned to our key question as to whether these activations, in addition to reflecting fluctuations in local confidence, also reflected information about global SPEs. We focused subsequent analyses on the key nodes of a local confidence network in the frontoparietal midline identified both in the current study and in previous literature: vmPFC, PRECU, and dACC (16, 21, 24, 25). As our behavioral analysis indicated that subjects’ global SPEs are sensitive to fluctuations in local confidence, we sought to examine whether activity in these three functionally defined regions of interest (ROIs) show evidence for encoding local confidence in a frame of reference sensitive to global SPEs.

Out of our three ROIs, we found that confidence-related activity in vmPFC and PRECU, but not dACC, differed between trials corresponding to higher and lower global SPEs (Fig. 2). Specifically, activity in vmPFC increased with confidence for lower (*β* = 0.97, *P* = 1.8e-04) but not higher SPE trials (*β* = 0.11, *P* = 0.74), with a significant difference between higher and lower SPE trials (*t*_38_ = −2.14, *P* = 0.039) (Fig. 2*D*). Similarly, activity in PRECU increased with confidence for both lower (*β* = 0.81, *P* = 4.5e-06) and higher (*β* = 0.37, *P* = 0.0179) SPE trials, and did so significantly more so for lower SPE trials (*t*_38_ = −2.32, *P* = 0.032) (Fig. 2*E*). In contrast, activity in dACC decreased with confidence on both higher (*β* = −0.99, *P* = 1.1e-05) and lower (*β* = −1.07, *P* = 9.5e-07) SPE trials to a similar extent (*t*_38_ = 0.32, *P* = 0.75, Bayesian *t* test BF = 0.18 indicating substantial evidence for a null hypothesis of no difference) (Fig. 2*F*). To establish the specificity of these results, we additionally searched at a whole-brain level for interactions between local confidence encoding and global SPEs, but no clusters survived correction for multiple comparisons. Taken together, these findings indicate that vmPFC and PRECU tracked local confidence in a manner that is sensitive to global SPEs.

### Brain Activity Tracking Global SPEs Irrespective of Local Confidence.

Our previous analyses focused on how the encoding of local confidence is itself modulated by, or interacts with, global SPEs. We next asked whether global SPEs are also tracked in a manner that is independent of local confidence (i.e., a main effect of global SPE). To examine this, we contrasted brain activity on trials that corresponded to higher as compared to lower SPEs, irrespective of trial-by-trial fluctuations in local confidence (*Materials and Methods*). Within our ROIs, we did not observe any overall differences in activity between higher and lower global SPEs in vmPFC (*t*_38_ = 1.18, *P* = 0.25) and PRECU (*t*_38_ = 1.31, *P* = 0.20) (Fig. 2 *G* and *H*). We found a difference in our broader dACC ROI (*t*_38_ = 2.16, *P* = 0.037), but this was no longer apparent when taking a sphere centered on the dACC peak voxel (*t*_38_ = 0.06, *P* = 0.95, and *SI Appendix*, Fig. S5*I*) or when computing this contrast at the whole-brain level (see below), and we do not interpret it further. In contrast, at a whole-brain level, we revealed stronger activity for higher SPEs in bilateral ventral striatum (left: *β* = 0.45, *P* = 4.73e-04; and right: *β* = 0.54, *P* = 3.86e-04, FWE-corrected for multiple comparisons at the cluster level) (Fig. 3*A*), and in two occipital areas (all *β* > 0.93, all *P* < 0.020, FWE-corrected for multiple comparisons at the cluster level) (*SI Appendix*, Table S1). No negative effect was found for this contrast (*SI Appendix*, Table S1). To further visualize this pattern, in a separate general linear model (GLM) we estimated activity in bins of local confidence separately for higher and lower SPE trials (Fig. 3*B*) (*Materials and Methods*).

### Relationship between Behavior and Neural Signals of Global SPEs.

**Fig. 4.**
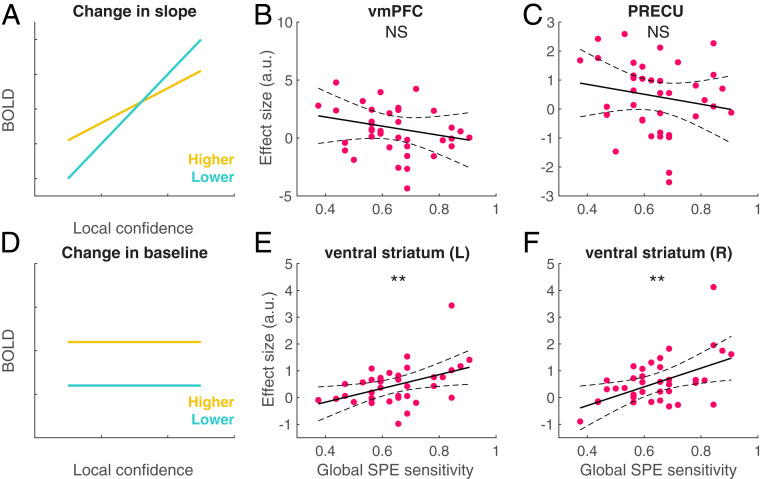
Brain–behavior relationships. (*A* and *D*) Schematic summary of fMRI findings in (*A*) vmPFC and PRECU and (*D*) ventral striatum. (*B* and *C*) No significant between-subjects correlation was found between a change in slope observed in vmPFC (*B*) or PRECU (*C*) and global SPE sensitivity, defined as the frequency of choosing the objectively easier of both tasks at the end of blocks. (*E* and *F*) A significant between-subjects correlation was found between neural effects of global SPE in left (*E*) and right (*F*) ventral striatal activity and global SPE sensitivity. Each circle corresponds to a participant. **P < 0.005. The dotted lines indicate 95% confidence intervals. See also *Materials and Methods* and *SI Appendix*, Table S1.

Together, our fMRI analyses reveal two forms of association between brain activity and global SPEs. First, we observed confidence-related activity that differed according to global SPE in vmPFC and PRECU (Fig. 2). Second, we observed a main effect of global SPEs in the absence of modulation by local confidence in ventral striatum (Fig. 3). In a final exploratory analysis, we sought to examine which (if any) of these effects was most predictive of subjects’ behavioral global SPEs. Here, we define global SPE sensitivity as the extent to which subjects selected the objectively easier task at the end of blocks. We tested two, nonmutually exclusive hypotheses. A first hypothesis is that subjects whose local confidence-related signals are more strongly modulated by global SPEs (“Change in slope,” Fig. 4*A*) would have greater global SPE sensitivity (Fig. 1*B*). A second hypothesis is that subjects who show a stronger overall encoding of global SPEs (“Change in baseline,” Fig. 4*D*) would have greater global SPE sensitivity (Fig. 1*B*).

Regarding our first hypothesis, we found no significant correlation between a change in slope in vmPFC (*ρ* = −0.20, *P* = 0.22) or PRECU (*ρ* = −0.19, *P* = 0.28) and global SPE sensitivity (Fig. 4 *B* and *C*). In contrast, when testing our second hypothesis, we found that the extent to which ventral striatal activity differentiated between higher and lower global SPE trials was significantly correlated with behavioral global SPE sensitivity (Fig. 1*B*) (left ventral striatum: *ρ* = 0.45, *P* = 0.0042; right ventral striatum: *ρ* = 0.47, *P* = 0.0027) (Fig. 4 *E* and *F*). These correlations remained significant when excluding one outlier subject observed in the scatter plots in Fig. 4 *E* and *F* (left ventral striatum: *ρ* = 0.38, *P* = 0.018; right ventral striatum: *ρ* = 0.40, *P* = 0.012).

## Discussion

Previous research in human and nonhuman primates has identified neural networks supporting the generation of local confidence judgements about simple decisions (12, 13, 17, 23). However, a major determinant of human behavior is not only local confidence, but also global SPEs about our skills and abilities, related to self-efficacy beliefs that are formed over longer timescales (4) and are thought to be altered in a number of mental health disorders (6, 9). Behaviorally, recent work identified a contribution of local confidence to the formation of global SPEs (3), but the neural underpinnings of a mapping from local to global confidence have remained unclear. Here, we examined whether local confidence-related brain activity is modulated by global SPEs using fMRI. Within a network of cortical areas associated with local confidence-related activity, vmPFC and PRECU additionally tracked local confidence in a manner that was sensitive to current global SPEs. In contrast, we observed that bilateral ventral striatum was more active for trials with higher as compared to lower global SPEs, irrespective of local confidence level. In what follows we discuss each of these key results in turn.

We were initially agnostic about which brain region, if any, might encode confidence in a manner that was sensitive to global SPEs, and had no hypothesis regarding the directionality of this relationship. Consistent with previous findings, we identified a network containing vmPFC and an area encompassing posterior cingulate cortex and PRECU in which activity was positively related to local confidence, whereas activity in a wider frontoparietal network was negatively associated with local confidence (17, 18, 21). These signals suggest that local confidence encoding may be quite generic (29), overlapping with regions identified as encoding confidence in value-based choices (22, 23), metamemory, and metaperception (25). Within ROIs selected for their encoding of local confidence, only activity in the vmPFC and PRECU clusters encoded it in a manner that was additionally modulated by global SPEs.

Specifically, in vmPFC and PRECU, we found stronger local confidence-related activity on trials that corresponded to lower global SPEs, as revealed by end-of-block task choices (Fig. 2). We did not observe any areas that showed the reverse effect (stronger confidence encoding on trials of higher SPEs) at the current analysis threshold. Although this pattern may initially appear surprising, we suggest two alternative interpretations for this encoding asymmetry. First, encountering an estimate of high local confidence may be more surprising in the context of a low global SPE task, leading to an increased neural response on such trials. More broadly, these activations could reflect a form of prediction error in relation to an internal model of task performance (30), possibly guiding internal decisions to switch tasks had they not been externally cued (31). In other words, when responding to the task on which we think we are performing better, a locally elevated confidence signal is consistent with our internal model, and no prediction error ensues. In contrast, a locally elevated confidence signal on a task we believe we are performing badly generates a positive prediction error. An alternative interpretation is that local confidence signals contribute to evidence accumulation across a block, akin to a drift-diffusion process but now occurring across instead of within trials (32). In our case, each local confidence signal can be thought of as a sample of evidence about the current task SPE. This interpretation is consistent with models of learning in which the brain accumulates evidence for switching away from a default option (33), and with computational models of SPE learning in which local confidence is used to update posterior beliefs over expected global task SPEs (3).

Both of these accounts suggest that local confidence should have symmetric effects on high and low SPE trials—i.e., we should also observe greater neural responses to low confidence in the context of a high SPE task, which was not the case in our data in which confidence selectively modulated activity on low SPE trials. Instead, we suggest that such asymmetries may be related to the nature of the information encountered under high and low SPE trials. Specifically, subjects may process information about the self differently according to whether it is positive or negative (34), with greater belief updating following good compared to bad news (35). Similarly in reinforcement learning tasks it has been shown that positive (better-than-expected) prediction errors are integrated more rapidly than negative prediction errors (36). In the context of our paradigm, low confidence in the context of a high global SPE is “bad” news about one’s performance, so such an update might be expected to be downweighted. In contrast, high confidence in the context of a low SPE is “good” news. The current findings also suggest an intriguing reinterpretation of local confidence activations reported in earlier work. Even when trials are designed to be independent, subjects often perform multiple trials of an experimental task, and may naturally build up global expectations about their performance over time. It is therefore plausible that global and local confidence signals both contribute to observed activations even in situations in which global confidence is not explicitly measured.

The activations we observed here in relation to local confidence, and which formed the basis of our vmPFC and PRECU ROIs, are consistent with those found in previous studies of metacognition. Previous work examining variation in activation location and sulcal morphology (e.g., ref. 37) has highlighted the potential for heterogeneous definitions of the vmPFC across studies. Here, our vmPFC cluster was mainly located anterior to the cingulate gyrus, with voxels in superior and middle frontal gyri, and extending ventrally into pgACC, consistent with previous studies that have identified ventromedial subregions of PFC as key nodes for confidence estimates across a range of tasks and cognitive domains (19–23, 33, 38). In addition to vmPFC, our PRECU ROI also encoded local confidence in a manner that discriminated between global SPEs (Fig. 2*E*). This ROI was located anterior to the calcarine sulcus and dorsal and anterior to the parieto-occipital sulcus, predominantly falling in the posterior cingulate gyrus. Interestingly, metamemory tasks have been shown repeatedly to engage a similar region, mostly its mid and posterior cingulate parts (ref. 39; for reviews, see refs. 29, 40, 41). Moreover, previous work using structural MRI has identified a relationship between PRECU gray matter volume and individuals’ metacognitive ability, in an area overlapping with our activation, albeit located more dorsally (24, 26). Similarly, multivariate analyses of fMRI data have revealed that it is possible to discriminate high- and low-confidence trials using PRECU activity across both perceptual and memory tasks (25). Together with the present study, these results suggest an involvement of vmPFC and PRECU across multiple hierarchical levels and domains of metacognitive evaluation. A possible explanation for both these and the present findings is that the PRECU is engaged when retrieving prior beliefs about global self-ability from memory, a functional role that transcends both local and global metacognitive evaluation. Both PRECU and medial PFC have long been implicated in the maintenance of self-related information, for instance in tracking self-generated action (42), retrieving memories about the self (43), or when inferring a causal link between internal intentions and actions as compared to a link between external events and their consequences (44). It is possible that maintaining and updating global SPEs requires similar access to memories for self-related skills and abilities.

In bilateral ventral striatum, we identified stronger activity on trials corresponding to higher as compared to lower SPEs, in the absence of parametric effects of local confidence (Fig. 3). We also found that subjects whose SPEs were more in line with objective task difficulty also showed stronger striatal encoding of global SPEs (Fig. 4 *E* and *F*). Ventral striatal activity has previously been implicated in tracking local “confidence prediction errors” on a categorization task (28) and on a perceptual learning task (2). In the latter study, the authors found that striatal activity tracked the expected accuracy from a model in which local confidence was used to update estimates of task performance across trials. Notably, a running estimate of expected accuracy over several trials is analogous to a global SPE, despite there being only a single task to monitor in Guggenmos et al.’s study.

The set of regions we found implicated in global SPE representations, namely, ventral striatum, vmPFC, and PRECU, are also part of a brain network implicated more broadly in subjective valuation (45). Indeed, the role of these regions in tracking global SPEs is consistent with encoding internal expectations of success. Subjects were incentivized to select the task on which they thought they performed best, such that by the end of the block there was a close relationship between the difference in global SPEs and an expectation of an easier task choice. However, we note that all of our analyses focused on fMRI measurements during the block, rather than at the end of blocks when participants were asked to make the global choice. The observation that both local confidence and global SPEs modulated activity in vmPFC and PRECU on such trials argues against an interpretation in terms of overall reward expectation. It is also notable that these signals were observed in the absence of any explicit reward outcome delivered during the experiment, and multiplexed both local and global aspects of subjective confidence. Instead, our findings fit well with a growing body of evidence implicating a similar network not only in subjective valuation, but in the generation of confidence judgments (22, 25), with tight links between valuation and confidence signals suggesting that being confident may itself be intrinsically valuable (23). Moreover, our findings are also consistent with a role for confidence estimates—particularly at the global, or task, level—in contributing to long-run expectations about success. For instance, two recent value-based decision-making studies dissociating the reward value of a choice from its contribution to a task goal revealed that the patterns of activity in vmPFC (46) and posterior cingulate cortex and ventral striatum (47) were more consistent with task goal monitoring over longer timescales than encoding of local reward expectations.

Behaviorally, we replicate our previous findings of a combined influence of fluctuations in difficulty level and objective performance on global SPEs (3). It is particularly striking that here, subjects were able to form sensible global SPEs in the absence of external feedback and without any requirement for a local confidence rating, an experimental situation more challenging than our previous study in which intermittent feedback was available (3). In the context of our previous study, regularly requiring local confidence judgements may encourage subjects to pause and engage in reflecting about their confidence (48, 49), potentially allowing it to contribute more strongly to global SPE formation. Here, we sought to minimize confidence reporting requirements in the scanner, instead using a model that used a weighted combination of performance variables to infer fluctuations in latent confidence. It might be that, had we collected explicit reports in the scanner, additional variance introduced in the formation of explicit reports may have also contributed to global SPEs. More research is needed to understand how requiring explicit confidence judgments and feedback may affect the construction of global SPEs.

In summary, our study provides initial evidence that confidence encoding in the human brain may operate across multiple hierarchical levels—spanning from local, trial-specific confidence estimates to global estimates of task ability or skill. The present work on the coexistence of signals for local and global confidence may help bridge a gap between the rich models of local confidence (e.g., ref. 12) and higher-level self-assessments that have hitherto remained poorly characterized at a computational and neural level. Moreover, from a clinical standpoint, the formation of beliefs about our abilities is likely to prove important in understanding the generation of maladaptive beliefs about self-efficacy and self-esteem (4, 5). From this perspective, an understanding of the neural underpinnings of global self-beliefs provides a first step toward the development of behavioral and neural interventions aiming at restoring appropriate self-beliefs.

## Materials and Methods

### Participants.

The study was approved by the University College London (UCL) Research Ethics Committee (Project ID: 8231/001). We recruited 47 human subjects who provided written informed consent. This sample size aimed at providing sufficient power to study interindividual differences in metacognitive ability. Eligibility criteria included an absence of neurological or psychiatric conditions, normal or corrected-to-normal vision, and an absence of color blindness. Subjects were instructed that they would receive a £28 base payment and up to £2 bonus according to their performance. All subjects were eventually paid £30 for their participation. To ensure data quality, a series of exclusion criteria were applied. Six participants were excluded for responding at chance level, missing more than 5% of trials and/or selecting games purely according to their favorite colors, leaving *n* = 41 participants (24 females [f]/17 males [m]; aged 19 to 40; mean, 24 y old) for behavioral data analysis. We additionally excluded two participants due to excessive head movement (scan-to-scan motion > 3 mm), leaving *n* = 39 participants for fMRI analysis (23 f/16 m; aged 19 to 40; mean, 25 y old).

### Calibration Phase.

An initial perceptual decision-making task was performed outside the scanner. On each trial, subjects were asked to judge which of two boxes contained more small squares. The objective was to determine each subject’s psychometric function for this discrimination task. Stimuli were grid images containing a number of black squares, modified from previous experiments in our laboratory (3). Each box contained a grid of 400 positions. One of the two grids was always half filled (200 squares), whereas the other grid contained more squares (200 + square difference). Subjects performed 200 trials of varying levels of square difference equally distributed across 10 levels chosen following pilot work (2, 5, 7, 9, 10, 12, 14, 18, 34, and 70 squares), and randomized across trials. The position of squares inside each grid and the location (left/right) of the grid with more squares were randomized. During the calibration phase, subjects received feedback about their judgment (“Correct” or “Incorrect”). In this phase of the experiment, a black fixation cue was presented for 1,000 ms followed by the grid stimuli for 300 ms. Participants had 1,500 ms to respond, after which feedback was displayed for 800 ms and a 500-ms intertrial interval (ITI) ensued. Subjects were offered a break in the middle of the calibration session. From the fit of a cumulative normal psychometric function, we extracted two levels of difficulty (i.e., square difference) for each subject corresponding to a performance of 70% and 85% correct (16). These levels were recorded for use in the fMRI experiment.

### fMRI Experiment.

Inside the scanner, subjects performed short learning blocks featuring random alternation of two “tasks.” Each trial started with a color cue signaling which of the two tasks was in play (Fig. 1*A*). Each task consisted of a perceptual discrimination as to which of two images contained more squares. In all blocks, one task was easy and the other was difficult, based on the square difference between left and right images determined in the calibration procedure. Critically, subjects never received feedback about their judgments. At the end of each block, subjects were asked to choose between the two tasks for a monetary bonus. They were instructed that their bonus winnings would depend on their performance at the chosen task, so they were incentivized to choose the task for which they thought they performed best.

Subjects performed 32 blocks of 12 trials each. Each block contained six trials of each task, presented in a pseudorandom order such that there were no more than three consecutive trials of the same task. The left/right location of the box with more squares was pseudorandomized per block and per task. The mapping between color cues and tasks was fully counterbalanced across subjects. Each trial started with a color cue presented for 1,200 ms indicating the task of the current trial (Fig. 1*A*). Stimuli were then presented for 300 ms, and subjects had 1,500 ms in total to give their response. Trials were separated by jitters drawn from a uniform distribution between 2,500 and 5,500 ms. Jitters were pseudo-randomized such that long jitters were not systematically associated with difficult trials and vice versa, and were balanced per run, per block, and per task. At the end of blocks, task choice trials were unspeeded. After subjects responded, a border around the chosen task was illuminated for 1,500 ms (Fig. 1*A*). Within scanning runs, blocks were separated by a 10-s break. Longer breaks were provided between scanning runs.

### Metacognition Task.

After the scanning session, subjects performed a metacognition task in order to allow 1) estimation of individuals’ metacognitive abilities and 2) build a confidence model for use in the analysis of in-scanner data (*Behavioral analyses*). Subjects performed 200 trials of the same perceptual discrimination task as in the calibration phase, but instead of receiving feedback, they were asked to give a trial-by-trial confidence rating in the correctness of their response, using a scale from 1 (relatively low) to 6 (relatively high confidence). Each trial started with a fixation cue (1,200 ms), followed by stimuli (300 ms). Subjects had 1,500 ms to give their perceptual response. They were then presented with a confidence scale and had 3,500 ms to give their confidence response, followed by an ITI of 500 ms. As in the fMRI experiment, two difficulty levels were employed targeting performance levels of 70% and 85% correct determined in the calibration procedure. Trials of the two difficulty levels were randomly interleaved, with the box with more squares being on the left (respectively, right) on half of trials.

### Behavioral Analyses.

#### fMRI experiment.

To first assess the effectiveness of our difficulty manipulation, we computed mean performance and reaction times (RTs) in easy and difficult conditions, and tested the difference using paired *t* tests. Missed trials and trials with log(RT) outside of 3 SDs from the mean were excluded from subsequent analyses (mean = 3.5% of trials across subjects). To establish whether end-of-block task choices, our index of global SPEs, were related to difficulty level, we examined how often subjects chose the easiest of both tasks. Furthermore, to investigate whether participants took into account fluctuations in performance, we split task choice data in blocks with a larger (respectively smaller) difference in performance between tasks and tested the difference in task choice frequency using paired *t* tests (note that in this analysis two subjects were removed for having no blocks in which they performed better on the more difficult task). We further examined whether accuracy, difficulty level, and their interaction influenced task choice using a logistic regression. We *z*-scored regressors to ensure comparability of regression coefficients.

#### Metacognition task.

We first computed mean performance, RTs, and confidence in easy and difficult conditions, and tested the difference using paired *t* tests. As in the fMRI experiment, missed trials and trials with log(RT) outside of 3 SDs from the mean were excluded from subsequent analyses (mean = 1.8% of trials across subjects). We also compared confidence ratings between correct and incorrect trials, and visualized confidence distributions for correct and incorrect trials separately. Finally, we quantified the influence of our experimental factors, Accuracy and Difficulty Level, on confidence ratings in a 2 × 2 ANOVA.

A first objective of the metacognition task was to estimate metacognitive ability for each participant. We first computed meta-*d′*, a metric based on signal detection theory (SDT) that evaluates the extent to which a subject’s confidence ratings discriminate between their correct and incorrect responses (50). Subject-specific meta-*d′* parameters were obtained by fitting confidence rating data using the HMeta-d toolbox (51). By dividing meta-*d′* by *d′*, we obtain metacognitive efficiency, our measure of interest, that represents metacognitive ability corrected for differences in performance. Because SDT assumes a constant stimulus strength, we estimated two meta-*d’* values separately for the two difficulty levels. We also computed a model-free index of metacognitive ability, the type-2 area under the receiver operating curve (AUROC2) per difficulty condition (50). Metacognitive efficiency and AUROC2 values were then each averaged across easy and difficult conditions to obtain two indices per subject. We also used a hierarchical Bayesian approach to estimate group-level meta-*d′/d′* values in each difficulty condition, obtaining satisfactory convergence values in both cases (both Ȓ = 1.0004) (51).

A second purpose of the metacognition task was to allow estimation of a regression model to predict trial-by-trial confidence ratings from features of the perceptual decision on each trial. We used an ordinal regression model to predict reported confidence (six levels) from difficulty level (difference in squares between left and right stimuli), accuracy and log(RTs), along with the interaction of difficulty and accuracy (*SI Appendix*, Fig. S2). Regressors were *z*-scored to ensure comparability of regression coefficients.

Regression coefficients were then taken to the group level and tested for significance (one-sample *t* test against zero). We formally compared this model to a set of alternative models in a systematic manner (*SI Appendix*, Fig. S2*A*), both quantitatively (by computing the deviance for each regression model) and qualitatively by visualizing the relationship between predicted and observed confidence on a subsample of trials for each subject (*SI Appendix*, Fig. S2*E*). We subsequently used the obtained regression coefficients to transform the behavioral variables recorded during the fMRI session (i.e., difficulty level [i.e., square difference], accuracy, and RTs) to return a predicted (inferred) confidence report for each trial, in an analogous approach to ref. 20.

Finally, we established that predicted confidence was meaningfully related to global SPEs assayed in the fMRI session by comparing the mean predicted local confidence between trials corresponding to higher and lower global SPEs, as revealed by end-of-block task choices (Fig. 1*D*). We also performed a logistic regression that sought to predict task choices from the difference in predicted confidence between tasks. We tested the significance of the regression coefficient across subjects using a one-sample *t* test.

### fMRI Acquisition.

For each subject, we acquired four runs of 255 fMRI volumes on a 3-T Siemens scanner with a 64-channel head coil at the Wellcome Centre for Human Neuroimaging, UCL, London. Functional images were acquired with the following parameters: repetition time (TR) = 3.36 s, echo time (TE) = 30 ms, 48 slices in sequential ascending order, voxel size = 3 mm^3^, and matrix size = 64 × 72. Slices were 30° tilted to minimize signal dropout around the orbitofrontal cortex. We also acquired structural images using a magnetization‐prepared rapid gradient‐echo sequence with the following parameters (52): voxel size, 1 mm^3^; 176 slices; TR = 2,530 ms; and TE = 3.34 ms; and field maps with 64 slices (ascending slice order, slice thickness, 2 mm and 1-mm gap), TE1 = 10 ms, TE2 = 12.46 ms, and in-plane field of view = 192 mm^2^ with resolution = 3 mm^2^.

### fMRI Preprocessing.

We preprocessed MRI data using SPM12 (https://www.fil.ion.ucl.ac.uk/). Images were first reoriented, and four dummy volumes were discarded at the beginning of each run. Standard preprocessing included slice-timing, realignment and unwarping using the field maps, coregistration, segmentation, normalization to MNI template with voxel interpolation = 2 mm^3^, and Gaussian spatial smoothing with full width at half-maximum = 8 mm. Motion parameters from the realignment and their first-order temporal derivatives were subsequently used as regressors of no interest in first-level analyses.

### Physiological Monitoring inside the Scanner.

Peripheral measurements of cardiac pulse and breathing were made together with scanner slice synchronization pulses using a Spike2 data acquisition system (Cambridge Electronic Design). The cardiac pulse signal was measured using an MRI-compatible pulse oximeter (model 8600 F0; Nonin Medical) attached to the subject’s finger. The respiratory signal (thoracic movement) was monitored using a pneumatic belt. A physiological noise model was constructed to account for artifacts related to cardiac and respiratory phase and changes in respiratory volume using an in-house MATLAB toolbox (53). Models for cardiac and respiratory phase and their aliased harmonics were based on RETROICOR (54) and a similar, earlier method (55). Basis sets of sine and cosine Fourier series components extending to the third harmonic were used to model physiological fluctuations. Additional terms were included to model changes in respiratory volume (56, 57) and heart rate (58). This procedure resulted in a total of 14 “biophysical regressors” that were sampled at a reference slice in each image volume to give a set of values for each time point. The resulting regressors were included as regressors of no interest in first-level analyses. Due to technical issues with recordings, four subjects did not have reliable physiological data. These regressors were therefore omitted in the first-level estimation for these subjects, but the first- and second-level contrasts were calculated similarly as for the other subjects.

### fMRI Analyses.

#### GLM.

In a standard whole-brain GLM each trial was labeled as corresponding to a higher or a lower SPE according to the task chosen at the end of the block, our proxy for global SPEs (Fig. 1). Higher and lower global SPE trials were then modeled as separate Dirac delta functions time-locked to the response onset and parametrically modulated by *z*-scored local confidence inferred from the behavioral regression model (see *Metacognition task* above):

BOLD∼β∗higher+β∗higher∗confidence+β∗lower+β∗lower∗confidence.

Note that both parametric modulations, for higher and lower SPE trials, presented a similar variance and range of confidence values (paired *t* tests across subjects for variance, *P* = 0.77; for range [i.e., maximum – minimum], *P* = 0.58), ruling out the possibility that confidence-related modulation on either trial type could spuriously capture more variance in BOLD activity. Missed trials, task choice trials, and outlier reaction time trials were assigned to a separate regressor. Regressors of no interest included motion parameter regressors and their derivatives (12 regressors) and 14 regressors derived from physiological monitoring. We modeled each session separately (four sessions). All regressors were able to freely compete for variance (no orthogonalization). Four contrast images were created: confidence-related activity pooled across higher and lower SPE trials (*SI Appendix*, Fig. S4), confidence-related parametric activity for higher SPE trials and lower SPE trials separately (Fig. 2 and *SI Appendix*, Fig. S5), and the main effect of global SPE (contrast of higher vs. lower SPE trials, irrespective of confidence) (Fig. 3). We extracted ROIs that survived *P* < 0.05 FWE cluster-corrected for a cluster-defining threshold of *P* = 0.001, uncorrected, based on the contrast of local confidence-related activity irrespective of global SPEs (*SI Appendix*, Table S1). Using a leave-one-out procedure, regression coefficients were then extracted for each ROI. Regression coefficients extracted in a sphere of 6-mm radius centered on the peak voxel instead of the full cluster-based ROI provided virtually identical results (*SI Appendix*, Fig. S5). In *SI Appendix*, Table S1, clusters larger than 3,900 voxels (7.8 cm^3^) are reported as separate subregions when visual inspection indicated the presence of distinct clusters. Significance was assessed at the group level (one-sample *t* tests against zero). Effect size comparisons between contrasts were examined using paired *t* tests.

#### ROI analysis of local and global effects.

We fitted another GLM for the purpose of visualizing local confidence and global SPE effects in ROIs identified from the initial GLM. We divided trials into those which correspond to higher and lower global SPEs, and further split these into three tertiles of local confidence (high, H; medium, M; low, L), such that all six bins had the same number of trials:

BOLD∼β∗higherH+β∗lowerH+β∗higherM+β∗lowerM+β∗higherL+β∗lowerL.

The remainder of the model specification was identical to the initial GLM. Effect sizes were plotted in ROIs identified from the initial GLM (Figs. 2 *G*, *H,* and *I* and 3*B*).

#### Brain–behavior relationships.

Metacognitive ability as evaluated using local confidence judgements varies substantially across individuals (10) and is related to structural brain differences (26). However, it remains unknown whether the accuracy of global SPEs also varies across subjects, behaviorally and neurally. We first examined, behaviorally, whether better local metacognitive efficiency was associated with more accurate global SPEs (i.e., selecting more often the easy task and/or the best-performed task at the end of blocks; see *SI Appendix*). In addition, we examined whether subjects whose global SPEs were more sensitive to variation in objective difficulty differed in the neural encoding of global SPEs. We focused on brain areas where activity was sensitive to global SPE level. Using correlation analyses, we tested two complementary hypotheses (Fig. 4 *A* and *D*). First, we examined whether a change in slope (difference in the modulation of local confidence-related signals in vmPFC and PRECU by global SPEs; Fig. 2), or a change in baseline activity (strength of encoding of global SPEs in ventral striatum; Fig. 3) was related to global SPE sensitivity, defined as the frequency of choosing the easier task at the end of blocks (Fig. 1*B*).

## Supplementary Material

Supplementary File

## Data Availability

MATLAB code and behavioral data for reproducing the main figures and statistical analyses of the study are freely available at https://github.com/marionrouault/LocalGlobal. Second-level maps for the contrast images of the study are freely available at https://neurovault.org/collections/6591/. Further requests can be addressed to M.R.
