# Supplementary material for: Formation of global self-beliefs in the human brain
Source: Proc Natl Acad Sci U S A. 2020 Oct 15;117(44):27268–76. doi: 10.1073/pnas.2003094117 (PMC7959580; doi:10.1073/pnas.2003094117)
Supplement: Supplementary File [file pnas.2003094117.sapp.pdf]

Supporting Information: Appendix

Formation of global self-beliefs in the human brain

Marion Rouault[1,2,*] and Stephen M. Fleming[1,3,4]

[1]*Wellcome Centre for Human Neuroimaging, University College London, 12 Queen Square, London WC1N 3AR, UK.*
[2]*Department of Cognitive Studies, Ecole Normale Supérieure, PSL Research University, 29 rue d'Ulm, 75005 Paris, France.*
[3]*Max Planck UCL Centre for Computational Psychiatry and Ageing Research, University College London, 10-12 Russell Square, London WC1B 5EH, UK.*
[4]*Department of Experimental Psychology, University College London, 26 Bedford Way, London WC1H 0AP, UK.*

[*]Correspondence: Marion Rouault

Email: marion.rouault@gmail.com

ORCIDs
Marion Rouault: 0000-0001-6586-3788
Stephen M. Fleming: 0000-0003-0233-4891

Supplementary Results

Subjects' behavior on the metacognition task

As expected, subjects performed better on easy (84% correct) as compared to difficult (69% correct) trials (t_{40}=-14.7, p<1e-20) (Fig. S1C) and responded faster on easy (mean=830ms) than difficult (mean=867ms) trials (t_{40}=7.7, p=2.0e-9) (Fig. S1D). Confidence ratings indicated that subjects held higher confidence on easy compared to difficult trials (t_{40}=8.9, p=1.8e-10), and on correct compared to error trials (t_{40}=12.1, p=6.7e-15), indicating a degree of metacognitive sensitivity (Fig. S2B). Accordingly, a 2 × 2 ANOVA revealed a significant main effect of accuracy (F(1,40)=118.9, p=1.5e-13) and of difficulty level (F(1,40)=9.1, p=.0044) on confidence ratings, together with a significant interaction between these factors (F(1,40)=15.3, p=3.5e-04), indicating that confidence was rated higher for easy than difficult trials, an increase that was more prominent for correct trials (Fig. S2C). Together these results indicate that subjects gave reliable confidence ratings as a function of our experimental parameters.

Regression models predicting confidence from the metacognition task

Our goal was to predict local confidence in the fMRI experiment from a model fitted to the metacognition task data collected outside of the scanner. We adopted this approach, rather than eliciting confidence ratings in the scanner, for several reasons. First, we were keen to avoid interrupting the process of learning global self-performance estimates (SPEs) by requiring trial-by-trial explicit confidence ratings. Second, it has become increasingly clear that the neural and computational processes involved in forming explicit ratings are likely to be at least partly distinct from those involved in the formation of latent or "implicit" confidence estimates. The former appears to engage anterior-lateral PFC (1, 2), which we interpret as supporting a mapping between a "latent" and an "explicit" estimate of confidence (3). It remains an open question as to whether such a transformation would impact the formation of global SPEs – and future work may wish to specifically investigate the extent to which implicit and explicit confidence representations differentially contribute to global SPEs. A final benefit of omitting explicit ratings in the scanner was the ability to obtain more trials per

scanning session. Had we had introduced trial by trial confidence ratings, the time needed for an individual trial would have been substantially increased.

Accordingly, we sought to build a regression model to infer trial-by-trial variations in local confidence in the absence of explicit reports in the fMRI experiment. First, we compared a set of regression models representing the various parameters of our experimental design that contributed to confidence reports in the metacognition task (Fig. S2A) (see also Methods). As expected, all models included accuracy (all β>0.27, all p<4.7e-11) and RTs (all β<-0.61, all p<4.4e-12) as significant predictors. We omitted lagged terms for the influence of previous confidence ratings as these could not be estimated in the absence of explicit reports in the fMRI experiment, despite finding that they improved the fit to the metacognition task data, in line with previous findings (4).

Quantitatively, as a criterion for arbitrating between models, we used deviance, a goodness-of-fit statistic reflecting model evidence for each regression model (as returned by Matlab's *mnrfit* function, with lower deviance indicating better model). We found that the best model was Model 3, which included additional predictors for difficulty (β=.11, p=.043) and the interaction between accuracy and difficulty (β=.14, p=.19), consistent with the results of our model-free ANOVA (as indicated by the lowest cumulated deviance=22487; in all other models, cumulated deviance>22548). Note that here we were not interested in penalising for model complexity or abitrating between different models, but only in predicting confidence as accurately as possible in the fMRI experiment. To establish whether this approach was able to identify idiosyncratic fluctuations in local confidence, for each subject s, we compute the predicted confidence under Model 3, either when fitted to the subject's own data ($s = i$), or fitted to each other subject i's data ($s \neq i$). We then computed the sum of squared error between predicted and reported confidence for each pairing of s and i. We found that each subject was consistently better predicted by the confidence model fitted on their own data ($s = i$) than on any other subject's data ($s \neq i$) (mean rank = 1). We also visually inspected the correspondence between observed and predicted confidence from our selected Model 3, confirming that the predictions were meaningful (Fig. S2E). We note that predicted confidence fluctuations were of smaller amplitude to

those measured in the metacognition task (Fig. S2E). Importantly however, the direction and amplitude of confidence fluctuations were properly captured, such that even if baseline confidence were altered by factors such as noise or stress inside the scanner, we were able to meaningfully identify confidence-related activations in a linear model of the BOLD response, in which parametric modulations were z-scored.

We also note that using confidence estimates extracted from Model 5 (second best-fitting model) gave very similar results. For instance, we found higher predicted confidence on trials associated with higher global SPEs (t_{40}=6.5, p=9.7e-08). We again found more confidence-related activity on trials associated with lower SPEs in vmPFC (p=1.22e-15, FWE-corrected for multiple comparisons at the cluster level) and PRECU (p=8.54e-10, FWE-corrected for multiple comparisons at the cluster level), and a main effect of global SPEs in bilateral ventral striatum (both p<4.2e-04, FWE-corrected for multiple comparisons at the cluster level), in line with results obtained using Model 3 (Fig. 1D, 2 and 3). The local confidence network identified here also closely matches that reported in previous work using in-scanner explicit confidence reports, supporting the validity of our approach (5, 6).

Metacognitive ability

Using the metacognition task, we quantified each subject's metacognitive efficiency (meta-*d'*/*d'*) and type-2 Area Under the Receiver Operating Curve (AUROC2) metrics (see Methods) for each difficulty level. Using a hierarchical fit, we found a metacognitive efficiency of 0.53 for easy and 0.67 for difficult trials, a difference that was not significant (HDI of the difference between easy and difficult trials overlaps with zero: [-0.325, 0.053]) (Fig. S3A). Averaging over easy and difficult conditions indicated a metacognitive efficiency of 0.60 and an AUROC2 of 0.62 (0.64 for easy trials and 0.60 for difficult trials, Fig. S3B), in line with previous studies of metaperception (5).

We found mixed relationships between local metacognitive ability estimated on the metacognition task and the accuracy of global task choices in the fMRI experiment. In the main text we define global SPE sensitivity with respect to objective difficulty level (i), but an alternative definition would be with

respect to performance fluctuations (ii). These two definitions of global SPE sensitivity were strongly correlated across subjects ($\rho=0.57$, $p=.0001$), suggesting a general capacity for “global” metacognition. While we found no correlation between global SPE sensitivity under definition (i) and metacognitive efficiency ($\rho=0.13$, $p=.43$) or AUROC2 ($\rho=0.17$, $p=.29$), when using definition (ii) we identified a significant correlation between the frequency of choosing the best-performed task and both metacognitive efficiency ($\rho=0.41$, $p=.007$) and AUROC2 ($\rho=0.32$, $p=.04$).

Supplementary Figures and Table

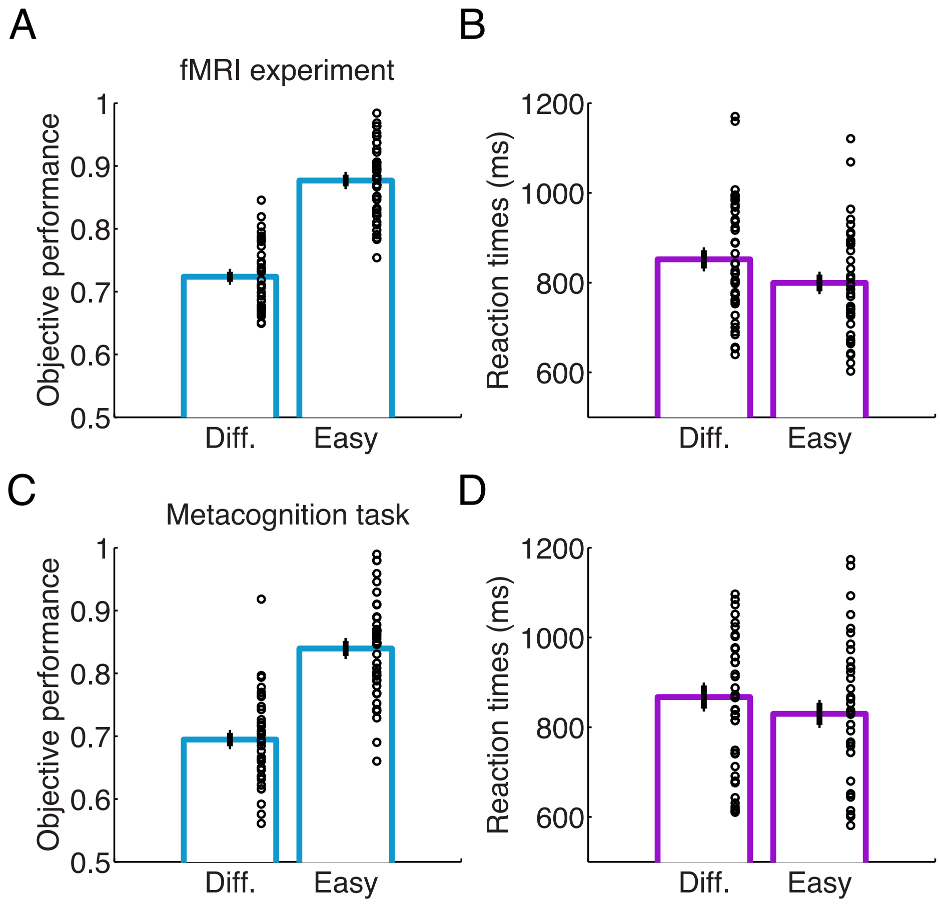

Figure S1. Subjects' behavior (N=41)
In the fMRI experiment, subjects performed better (A) and responded faster (B) on easy compared to difficult trials. Similarly in the metacognition task, subjects performed better (C) and responded faster (D) on easy compared to difficult trials. Error bars represent S.E.M. over participants and black circles represent individual data points.

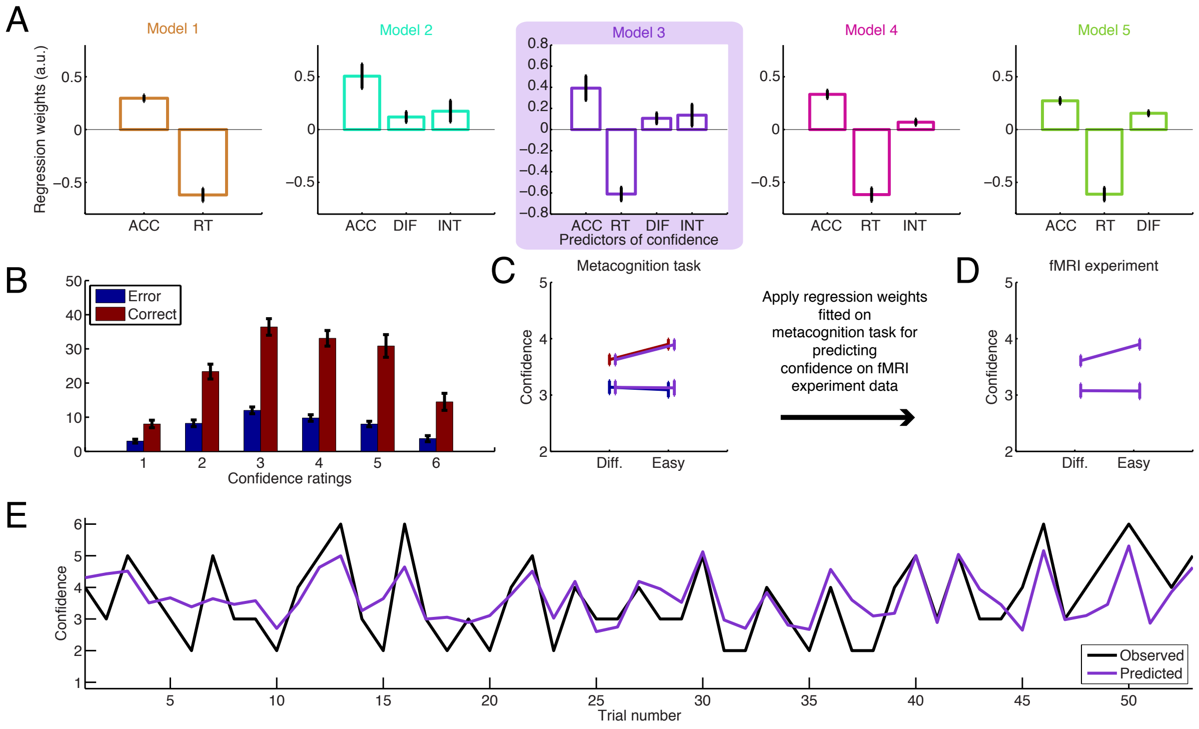

Figure S2. Subjects' behavior in the metacognition task outside of the scanner (N=41)
A) Set of five regression models predicting confidence. Predictors are accuracy (ACC), reaction times (RT), difficulty level (DIF) and the interaction between accuracy and difficulty level (INT). B) Confidence distributions indicating that subjects gave higher confidence ratings for correct (red) than error (blue) trials. C) Confidence increased for easy as compared to difficult trials, and for correct as compared to error trials (see Results), both for subjects (red/blue) and Model 3 predictions (purple). D) Predicted confidence in the fMRI experiment according to Model 3 (purple) fitted on the metacognition task data, for correct (upper line) and incorrect (lower line) trials. Error bars represent S.E.M across subjects. E) Predicted confidence from selected Model 3 on a subset of trials (purple) for an example subject, plotted together with their confidence responses in the metacognition task (black) (for illustration purposes).

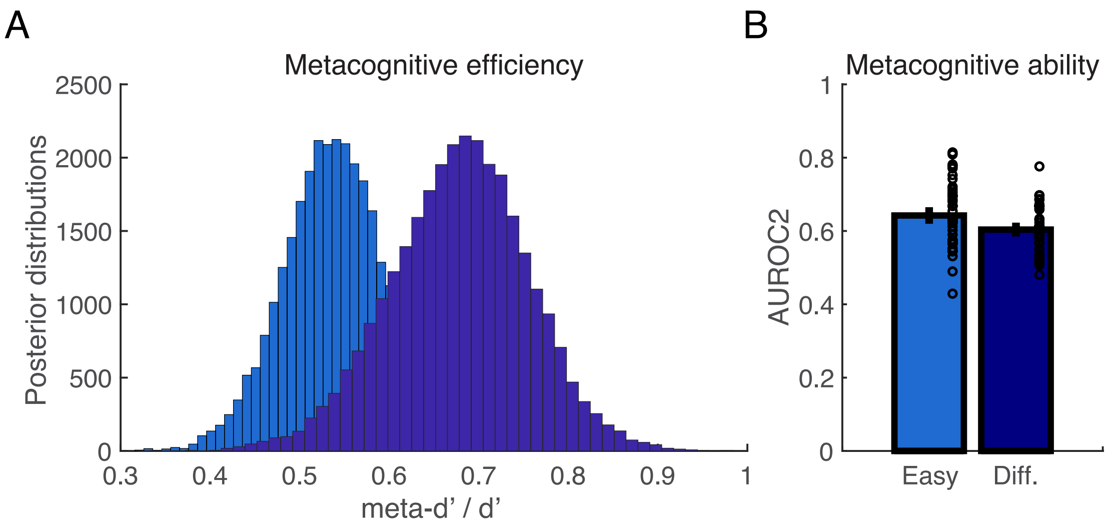

Figure S3: Metacognitive ability assessed on the metacognition task outside of the scanner. A) Posterior distributions over hierarchical Bayesian estimates of metacognitive efficiency (meta-d'/d'; light blue: easy condition; dark blue: difficult condition). B) Type-2 Area Under the Receiver Operating Curve (AUROC2), estimated separately for easy (light blue) and difficult (dark blue) conditions. Error bars represent S.E.M across subjects and black circles represent individual data points (N=41 subjects).

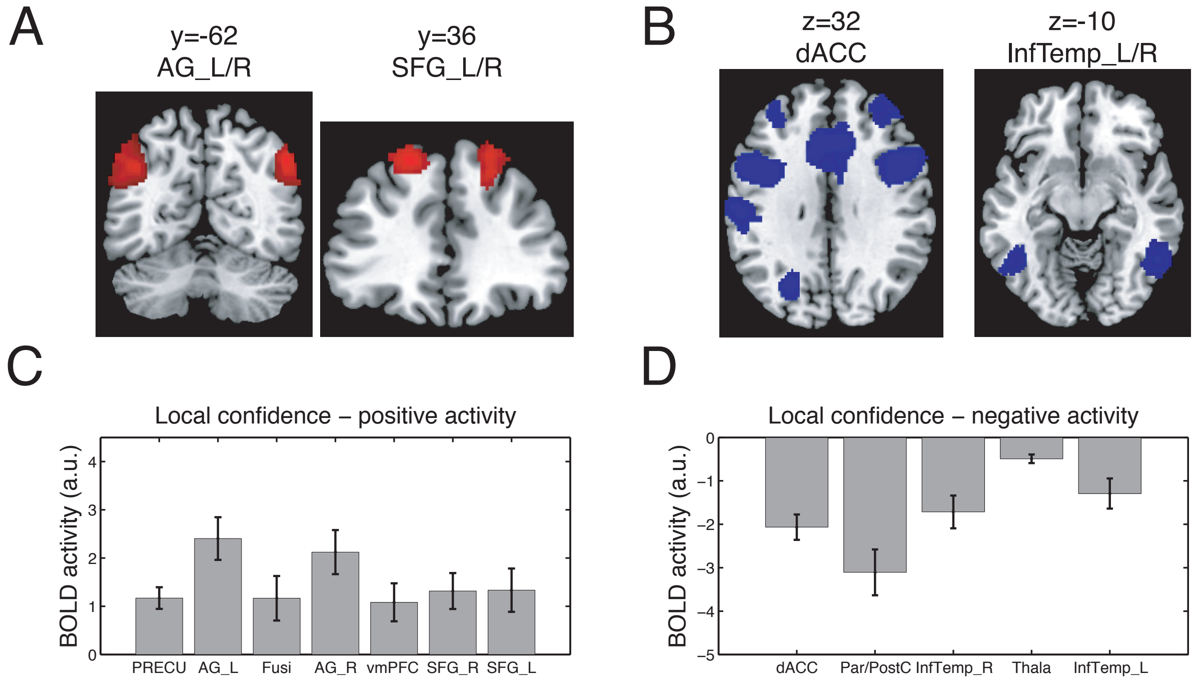

Figure S4: Local confidence-related activity irrespective of global SPEs (i.e. collapsed across higher and lower SPE trials). Positive (A) and negative (B) activations thresholded at p<.05 FWE cluster-corrected at a cluster-defining threshold of p=.001 uncorrected, superimposed on an anatomical template. MNI coordinates of the slice are indicated. For illustration purposes, only part of the network is illustrated on maps corresponding to activity thresholded at p=.005 uncorrected. (C, D) Regression coefficients for regions identified related to local positive (C) and negative (D) confidence activity, including the subset of regions of interest presented in (A) and (B). Regression coefficients were extracted in each ROI using a leave-one-out procedure. Error bars represent S.E.M. across subjects (N=39). See also Table S1. Abbreviations: AG, angular gyrus; SFG, superior frontal gyrus; dACC, dorsal anterior cingulate cortex; InfTemp, inferior temporal cortex; PRECU, precuneus; Fusi, fusiform gyrus; vmPFC, ventromedial prefrontal cortex; Par/PostC, postcentral gyrus and parietal cortex; Thala, thalamus. L, left; R, right.

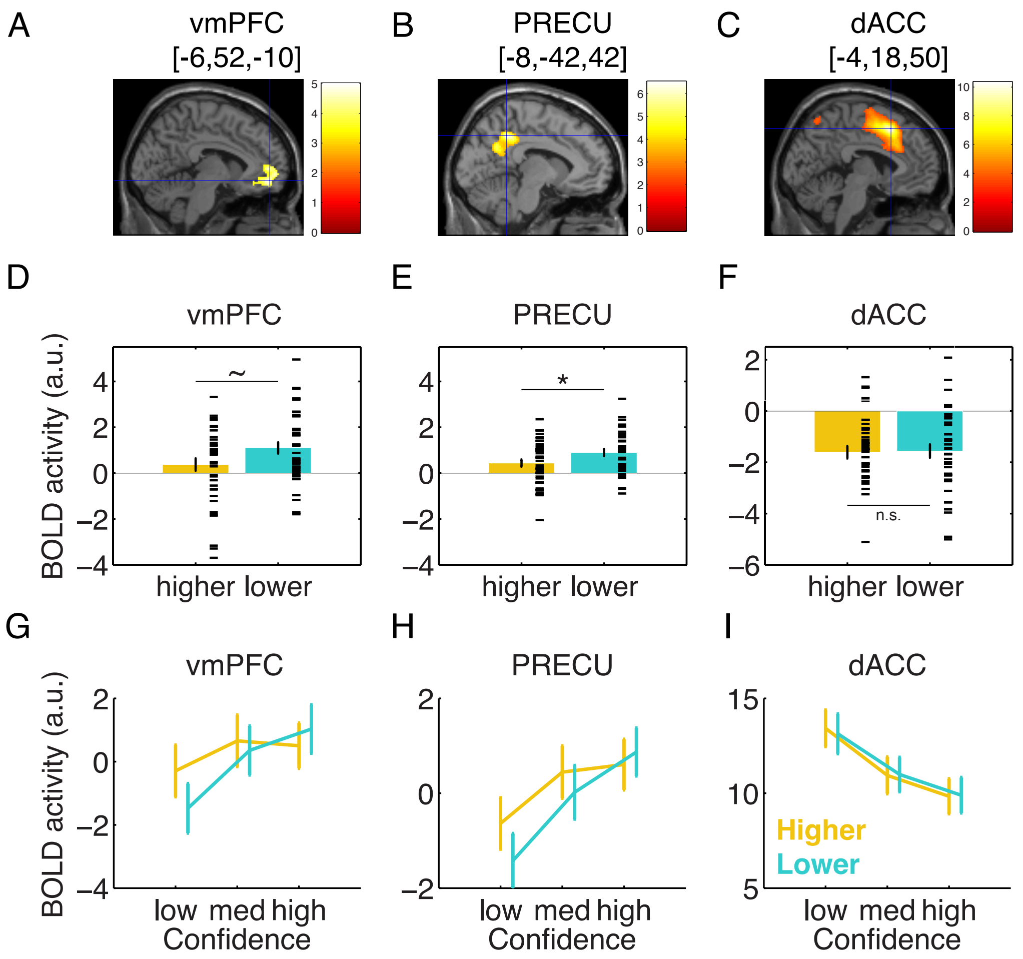

*Figure S5: ROI analysis of interactions between local confidence and global SPEs. Identical to main Figure 2, but activations are now plotted in a sphere of 6 mm radius centered on the peak voxel of each ROI. Error bars represent S.E.M. across subjects (N=39). Horizontal ticks indicate individual data points. *p<.05, ~p=.06, n.s. non significant, paired t-tests.*

Regions	Later.	pFWE-corr cluster-level	Peak MNI x	y	z	T (peak)	Size (voxels)
Positive effect of local confidence for higher SPE trials							
Fusiform gyrus	R	<.05	30	-80	-4	4.75	191
Angular gyrus (BA39)	L	<.05	-40	-68	42	4.48	210
Negative effect of local confidence for higher SPE trials							
Precentral gyrus (BA4),<br>Middle frontal gyrus (BA6),<br>Postcentral gyrus (BA3)	L	<.0001	-34<br>-32<br>-38	-20<br>-2<br>-28	56<br>54<br>52	-8.90	10166
Supramarginal gyrus (BA40)	R	<.0001	40	-34	46	-7.16	2862
Insula	L	<.0001	-34	18	8	-6.15	667
Insula, Inferior frontal cortex (BA9)	R	<.0001	46	10	30	-5.67	1777
Inferior temporal cortex (BA19)	R	<.01	54	-66	-6	-4.68	306
Positive effect of local confidence for lower SPE trials							
Mid- and Posterior cingulate cortex, Precuneus (BA31)	N/A	<.0001	-8	-42	42	7.65	1631
Inferior parietal lobule, Angular gyrus (BA19)	L	<.0001	-40	-72	38	5.64	852
Superior and middle frontal gyrus (BA8) (several peaks), vmPFC	L	<.0001	-22<br>-28<br>-6	30<br>24<br>58	50<br>50<br>-4	5.43	3944
Angular gyrus	R	<.005	50	-62	36	5.08	313
Mid-temporal cortex (BA21)	L	<.05	-62	-16	-14	4.57	188
Negative effect of local confidence for lower SPE trials							
Supplementary motor area,<br>Inferior parietal lobule,<br>dACC, mid-cingulate (BA9)	N/A	<.0001	10<br>-42<br>10	16<br>-36<br>26	50<br>48<br>36	-8.64	13927
Superior and Inferior parietal cortex, postcentral gyrus	R	<.001	34	-46	46	-7.29	3594
Insula, Inferior frontal cortex	L	<.0001	-28	20	10	-7.24	1100
Mid- and Inferior temporal gyrus	R	<.005	54	-54	-14	-4.78	293
Positive effect of contrast higher against lower SPE trials, irrespective of local confidence							
Ventral striatum	L	<.001	-18	10	-4	5.67	440
Ventral striatum	R	<.001	16	14	-4	5.43	453
Superior and middle occipital cortex (BA18-19)	L	<.005	-38	-86	30	4.77	315
Superior and middle occipital cortex (BA18-19), precuneus	R	<.05	34	-86	32	4.29	179
Precuneus / parietal cortex	L	<.05	-14	-60	48	4.18	192

Table S1: Regions with significant whole-brain activity thresholded at p<.05 FWE cluster-corrected at a cluster-defining threshold of p=.001 uncorrected for each contrast (see Methods). Later., laterality; L, left; R, right. BA: Brodmann area. Cerebellar activations were not analysed and are not reported here. No regions survived correction for the contrast lower > higher SPE trials.

References

1. Fleming SM, Huijgen J, Dolan RJ (2012) Prefrontal Contributions to Metacognition in Perceptual Decision Making. *Journal of Neuroscience* 32(18):6117–6125.

2. Gherman S, Philiastides MG (2018) Human VMPFC encodes early signatures of confidence in perceptual decisions. *eLife* 7:e38293.

3. Bang D, Ershadmanesh S, Nili H, Fleming SM (2020) Private–public mappings in human prefrontal cortex. *eLife* 9:415–25.

4. Rahnev D, Koizumi A, McCurdy LY, D'Esposito M, Lau H (2015) Confidence Leak in Perceptual Decision Making. *Psychological Science* 26(11):1664–1680.

5. Morales J, Lau H, Fleming SM (2018) Domain-General and Domain-Specific Patterns of Activity Supporting Metacognition in Human Prefrontal Cortex. *J Neurosci* 38(14):3534–3546.

6. Lebreton M, Abitbol R, Daunizeau J, Pessiglione M (2015) Automatic integration of confidence in the brain valuation signal. *Nat Neurosci* 18(8):1159–1167.
